# Toward personalizing treatment for depression: predicting diagnosis and severity

**DOI:** 10.1136/amiajnl-2014-002733

**Published:** 2014-07-02

**Authors:** Sandy H Huang, Paea LePendu, Srinivasan V Iyer, Ming Tai-Seale, David Carrell, Nigam H Shah

**Affiliations:** 1 Stanford Center for Biomedical Informatics Research, Stanford University, Stanford, California, USA; 2 Palo Alto Medical Foundation Research Institute, Palo Alto, California, USA; 3 Group Health Research Institute, Seattle, Washington, USA

**Keywords:** electronic health records, personalized medicine, depression, data mining, ontology

## Abstract

**Objective:**

Depression is a prevalent disorder difficult to diagnose and treat. In particular, depressed patients exhibit largely unpredictable responses to treatment. Toward the goal of personalizing treatment for depression, we develop and evaluate computational models that use electronic health record (EHR) data for predicting the diagnosis and severity of depression, and response to treatment.

**Materials and methods:**

We develop regression-based models for predicting depression, its severity, and response to treatment from EHR data, using structured diagnosis and medication codes as well as free-text clinical reports. We used two datasets: 35 000 patients (5000 depressed) from the Palo Alto Medical Foundation and 5651 patients treated for depression from the Group Health Research Institute.

**Results:**

Our models are able to predict a future diagnosis of depression up to 12 months in advance (area under the receiver operating characteristic curve (AUC) 0.70–0.80). We can differentiate patients with severe baseline depression from those with minimal or mild baseline depression (AUC 0.72). Baseline depression severity was the strongest predictor of treatment response for medication and psychotherapy.

**Conclusions:**

It is possible to use EHR data to predict a diagnosis of depression up to 12 months in advance and to differentiate between extreme baseline levels of depression. The models use commonly available data on diagnosis, medication, and clinical progress notes, making them easily portable. The ability to automatically determine severity can facilitate assembly of large patient cohorts with similar severity from multiple sites, which may enable elucidation of the moderators of treatment response in the future.

## Background and significance

Depression is one of the most prevalent psychiatric disorders, affecting about 14% of individuals worldwide. An estimated 10–20% of primary care visits are related to depression, making it the second most common chronic disorder seen by primary care physicians.^1^ ^2^ The economic cost of depression is staggering; in the USA, recent estimates put the direct expenses and loss of productivity resulting from depression at about $44 billion per year.^3^

Despite the prevalence of depression, treating it is a challenge. Many depressed patients are not even diagnosed: a meta-analysis performed by Mitchell *et al*^1^ found primary care physicians, who deliver the majority of care for depression, only identify about 50% of true depression cases. Improving diagnosis of depression would benefit patients, since early recognition and initiation of treatment is associated with a better prognosis, especially in patients exhibiting their first depressive episode.^4^ ^5^

Once a depressed patient is diagnosed, selecting the treatment modality is the next challenge. Although the different depression treatments have comparable average effectiveness, individuals vary widely in their response to treatment.^6^ The Sequenced Treatment Alternatives to Relieve Depression (STAR*D) study found only 36.8% of depressed patients experienced remission of depression symptoms after the first treatment. Patients were given the option of up to three additional treatments, resulting in an overall cumulative remission rate of 67%.^7^ However, most patients treated for depression, especially in primary care, undergo only one type of treatment: just 25% of patients pursue additional treatment options beyond their initial treatment.^6^ A major reason for this is that patients may not be willing to consider alternative modalities following an initial therapy they perceive to be ineffective.^8^ Thus, while customizing follow-up treatments can surely affect prognosis, it would also be very beneficial to personalize treatment for depression by selecting an initial treatment based on the characteristics of a given patient. In this regard, we are particularly interested in moderators—patient characteristics that predict differential treatment response—so that we might optimize initial treatment. Such moderators may include macroscopic and genomic biomarkers, as well as environmental factors. In our study we focus on the following potential moderators: patient demographics (eg, gender), diagnoses, prescriptions, procedures, and terms used in clinical text.

Simon and Perlis^6^ emphasize the difference between personalizing treatment for depression and clinical comparative effectiveness studies. The latter investigates the average effects of different treatments, while the former looks for moderators. For example, comparative effectiveness studies may indicate that drug A has a higher success rate than drug B for treating depression, but a given patient may have better results with drug B after accounting for moderators. Personalizing treatment for depression focuses on identifying such moderators.

We first use electronic health record (EHR) data to predict whether patients will be diagnosed with depression, and to determine how early a prediction can be made. To our knowledge, no such predictive models have been reported in the scientific literature. With good sensitivity at a high level of specificity, such a model could be used for early screening of patients; patients classified as having a high risk can be examined further. In addition, such a model could enhance cohort building for clinical studies on depression. Several investigators have demonstrated the utility of using EHR-derived features for automated cohort building.^9–12^

We also develop a model for assessing severity at initial treatment because baseline severity is closely linked with the effectiveness of treatment of depression. Patients with severe depression exhibit lower remission, regardless of treatment.^7^ ^13^ Prior work also suggests that patients with severe depression are more likely to benefit from medication as opposed to a placebo, whereas patients with mild depression respond approximately equally well to both.^14^ ^15^ Thus, assessing the initial severity of depression can assist in both selecting treatment and providing a baseline for evaluating the effectiveness of treatment.^16^

We also investigate the potential for using EHR data for personalizing treatment, by attempting to identify moderators that predict differential response to medication versus psychotherapy.

## Materials and methods

### Data sources and preprocessing

**Figure 1:**
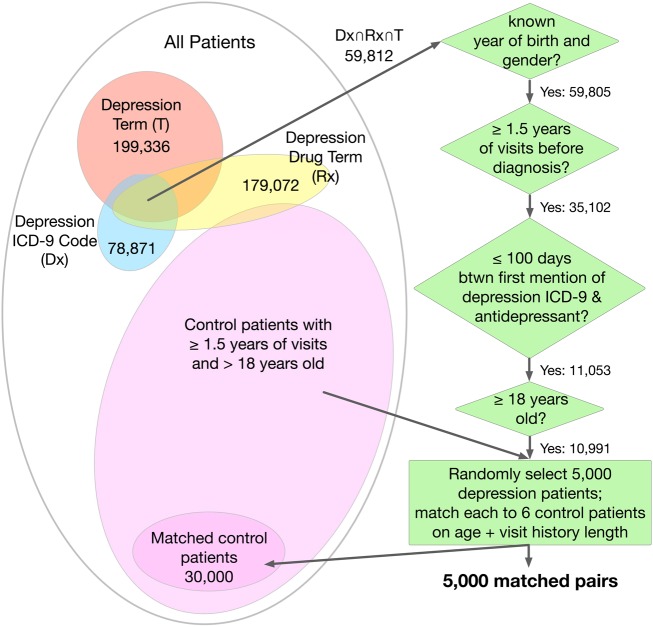
Selection of depression and control cohorts from the Palo Alto Medical Foundation (PAMF) dataset. ICD-9, International Classification of Diseases, Ninth Revision.

We used EHR data from the Palo Alto Medical Foundation (PAMF) and Group Health Research Institute (GHRI), both of which use the Epic EHR system. From the 1.16 million patients in the PAMF dataset, we selected 5000 depressed patients and 30 000 non-depressed patients (see the ‘PAMF cohort definition and validation’ section and figure 1). From the 600 000 patients in the GHRI dataset, we extracted a subset of 5651 patients treated for depression who have been scored using the Patient Health Questionnaire (PHQ-9) both at the start of treatment and after 90 days of treatment. The PHQ-9 is used for screening, diagnosing, and assessing the severity of depression; it comprises nine questions that are each worth three points, for a total score ranging from 0 to 27, divided into bins from minimal to severe depression (table 1).

**Table 1:** Baseline depression severity in the GHRI dataset, based on PHQ-9 score

PHQ-9 score	Depression severity	Number of GHRI patients
0–4	Minimal depression	267
5–9	Mild depression	747
10–14	Moderate depression	1294
15–19	Moderately severe depression	1652
20–27	Severe depression	1301

We only consider baseline PHQ-9 scores from patients’ first treatments.

GHRI, Group Health Research Institute; PHQ-9, Patient Health Questionnaire.

The following patient data from PAMF and GHRI are used: demographic data such as age and gender; structured data such as International Classification of Diseases, Ninth Revision (ICD-9) diagnosis codes, RxNorm prescription codes, and Current Procedural Terminology (CPT) procedure codes; and unstructured data such as progress notes, pathology reports, radiology reports, and transcription reports. All structured and unstructured data are time-stamped. Table 2 summarizes the overall characteristics of the PAMF and GHRI datasets and patient cohorts, including data types used and quantity of each type.

**Table 2:** Summary of PAMF and GHRI datasets

	PAMF	GHRI
Total patients	1.16 million	600 000
Cohort subset (% depressed)	35 000 (14.3%)	5651 (100%)
Gender split (% female)	55.2%	70.3%
Average follow-up time*	8.02 years	2.50 years
No. cohort visits (encounters)	1.18 million	226 000
Demographic variables		
Age	Included	Included
Gender	Included	Included
Ethnicity	Included	Included
Year of birth	Included	Included
Total structured data	2.34 million	1.50 million
ICD-9 diagnosis codes	2.34 million	521 000
CPT procedure codes	–	663 000
NDC prescription codes	–	310 000
PHQ-9 scores	–	5651
Total unstructured data	2.2 million	237 000
Radiology reports	Included	–
Pathology reports	Included	–
Transcription reports†	Included	Included

*Follow-up time is defined as the time between the first and last visit.

†Transcription reports include: progress, consultation, and nursing notes; secure messages, letters to patients, ER reports, discharge summaries, and other documents.

CPT, Current Procedural Terminology; ER, emergency room; GHRI, Group Health Research Institute; ICD-9, International Classification of Diseases, Ninth Revision; NDC, National Drug Code; PAMF, Palo Alto Medical Foundation; PHQ-9, Patient Health Questionnaire.

For the GHRI data, ICD-9 codes, RxNorm codes, and CPT codes are normalized to Concept Unique Identifiers (CUIs) from the Unified Medical Language System (UMLS) Metathesaurus. We processed the unstructured clinical texts from PAMF and GHRI as described previously in LePendu *et al.*^17^ In brief, we use an optimized version of the NCBO Annotator with a set of 22 clinically relevant ontologies (given in online supplementary materials, List 1), remove ambiguous terms,^18–20^ and flag negated terms and terms attributed to family history to reduce the false positive rate.^21^ The output of the annotation process is a mapping from each note to the terms in that note, including whether each term is flagged as negated and/or related to family history. Finally, terms are normalized to CUIs and are aggregated based on the hierarchies of the ontologies. Drugs are normalized to their active ingredients using RxNorm.^22^

The 35 000 patients (depressed plus non-depressed) from PAMF are used to train and test our model for predicting a future diagnosis of depression. The 5651 patients from GHRI are used to train and test our models for predicting treatment outcome and assessing severity; because the dataset contains PHQ-9 scores before treatment and after 90 days of follow-up, it provides a uniform measure for relative improvement. Moreover, because the diagnosis of depression is validated in the GHRI patients, we do not perform the selection and validation steps applied to the PAMF cohort.

#### PAMF cohort definition and validation

To train and test a model for diagnosing depression, we need a cohort of depressed patients and a control cohort of non-depressed patients. The depressed patient cohort consists of all patients over 18 years of age who meet criteria (explained below) ensuring that they: (i) indeed have depression, (ii) have a substantial medical history, and (iii) are likely diagnosed with the disorder at some point in their recorded medical history at PAMF (figure 1).^23^

To meet the criteria for having depression, patients must have: (i) a depression-related ICD-9 code (table 3),^24^ ^25^ (ii) a depression disorder term in their clinical text (online supplementary materials, List 2 gives the terms), and (iii) an anti-depressive drug ingredient term in their clinical text (online supplementary materials, List 3 gives the drug terms). The terms should not be negated or related to family history.

**Table 3:** Selected ICD-9 codes for depression

ICD-9 code	Description
296.2[0–6]	Major depressive disorder, single episode
296.3[0–6]	Major depressive disorder, recurrent episode
296.82	Atypical depressive disorder
298.0	Depressive type psychosis
300.4	Dysthymic disorder
311	Depressive disorder, not elsewhere classified

ICD-9, International Classification of Diseases, Ninth Revision.

For selecting the depression related terms, we start with a small seed set (including ‘depressive disorder,’ ‘major depressive disorder,’ ‘depression adverse event,’ and ‘depressive disorder, nec’) and expand this set to include terms subsumed by the seed terms via an ‘is-a’ relationship, and repeat the expansion again with the new set of terms. The final set of terms is manually filtered to remove terms that result in a significant number of false positives (ie, patients who have the term in their clinical text but did not have depression). Depression drug ingredients are selected by first retrieving drugs that treat depression from the Medi-Span (Wolters Kluwer Health, Indianapolis , Indiana, USA) Drug Indications Database, mapping them to active ingredients using RxNorm,^22^ and finally filtering out those active ingredients that are also present in drugs with primary indications other than depression.

A patient is considered to have a substantial medical history if the patient has at least 1.5 years of visits before the diagnosis. For each depression patient, we define the time of ‘first diagnosis’ as the first date in the patient's medical history at which both a depression-related ICD-9 code and an anti-depressive drug ingredient have occurred.^23^ This requirement of a substantial medical history ensures the model has enough data to make a prediction for each patient regarding diagnosis.

Finally, patients must have 100 or fewer days between the first mention of a depression ICD-9 code and an anti-depressant term in their medical history. This heuristic eliminates most patients who were diagnosed with depression before their medical history at PAMF began.

A total of 10 991 PAMF patients meet these criteria, and are thus included in the depression cohort (figure 1). To create the control cohort, we matched six randomly selected non-depressed patients to each of 5000 depressed patients randomly selected from the 10 991 patients in the depression cohort. We found using a subset of 5000 depressed patients greatly increases computational efficiency while not significantly harming model performance. This 1:6 matching ratio mirrors the 14% prevalence of depression in the general population and is within the range seen in primary care.^1^ ^26^ Control patients are patients having neither a depression-related ICD-9 code nor a depression term mentioned in their medical history. We match on age (in years) and visit history length (by 6-month periods). Matching patients based on length of visit history reduces the likelihood that the lack of a depression diagnosis in the control patient's medical record is due to inadequate medical record length, rather than the patient's actual lack of depression.

All 5000 depression patients were successfully matched to controls, resulting in a control cohort of 30 000 PAMF patients. To validate the quality of the depression and control cohorts, an expert in mental health conducted a manual review of the clinical text histories of 100 randomly sampled patients from the depression cohort (with 5000 patients), and of 100 randomly sampled patients from the control cohort. From this manual review, the estimated precision for the depression cohort is 96%, and the estimated precision for the time of first diagnosis is 79%. A time of first diagnosis is considered ‘correct’ if it is within 3 months of the actual time the patient was diagnosed with depression. The estimated precision for the control cohort is 91%. Ten of the 100 control patients were excluded from the analysis because they did not have adequate clinical notes to determine whether they had depression or not.

### Model creation and validation

We train logistic regression models for (i) predicting a diagnosis of depression, (ii) predicting response to treatment, and (iii) assessing severity of depression. Specifically, we use Least Absolute Shrinkage and Selection Operator (LASSO) logistic regression from the R glmnet package. LASSO is a type of logistic regression that uses L1 regularization, which penalizes the absolute value of the regression coefficients. This results in more coefficients equal to zero and thus more interpretable models.^27^ Since our model must handle thousands of features, LASSO is a good choice for pruning features that do not add predictive value. In contrast, L2 regularization, the other popular choice for penalized logistic regression, shrinks coefficients of less predictive features but not necessarily completely to zero. The features used from PAMF are gender, average number of visits per year, ICD-9 codes, and disease and drug ingredient terms in the annotated notes. We use similar features from GHRI: gender and CUIs from clinical text and coded data (consisting of diagnoses, prescriptions, and procedures), as well as age and baseline PHQ-9 score.

We exclude those features used for defining the depression cohort (see below), as well as terms flagged as negated and/or related to family history. ICD-9 codes, annotated terms, and CUIs are converted to binary features: 1 if the feature is present in a given patient's medical history and 0 otherwise. Average number of visits per year is calculated as average number of visits per day, multiplied by 365. This method gives more weight to a patient with only two visits a month apart, as opposed to a patient with only two visits a year apart. To convert average number of visits per year into discrete features, we use equal width interval binning, except the last bin has no upper bound. Equal width interval binning has been shown to produce results comparable to that of state-of-the-art supervised methods.^28^ ^29^ The visit bins are [0, 2), [2, 4), …, [18, ∞) in units of visits per year.

### Predicting a future diagnosis of depression

Our LASSO model for predicting diagnosis of depression classifies a given patient as having either a low risk or a high risk of an impending diagnosis of depression. In order to simulate the early prediction of a diagnosis of depression, the EHR data of patients are truncated at three different cutoffs: the time of ‘first diagnosis’ (for depressed patients) or the end of the medical history (for control patients), and 6 months before and 1 year before first diagnosis. We train the model on a randomly selected 80% of the 5000 depressed patients and their corresponding matched control patients, and test the model on the remaining 7000 patients. For the training data, we include EHR data until the first cutoff for depressed patients and include all available EHR data for controls, resulting in around 10 600 unique features. After training, we test the model on three different test sets: one for each of the three cutoffs defined previously. Thus, an accurate prediction of a depression diagnosis by the classifier, using EHR data up to the 6-months point, would be half a year earlier than the doctor's diagnosis of depression in the patient. Similarly, an accurate prediction using EHR data restricted to the time of first diagnosis would be analogous to the doctor's diagnosis.

### Predicting response to treatment

We attempt to predict response to treatment for depressed patients using a LASSO model. Specifically, we try to differentiate response for two treatment modalities: medication and psychotherapy. For each treatment modality, we train a model that predicts the likelihood a patient's depression will improve after they undergo the given treatment. Improvement is defined as a decrease of at least five points in the PHQ-9 score (which results in a change of severity level). We only consider the first treatment: 2472 patients are first treated with medication, 1576 of whom improved (63.8%); 2401 patients are first treated with psychotherapy, 886 of whom improved (36.9%). We exclude patients who received both medication and psychotherapy for their first treatment, since we are interested in differential response to one treatment versus the other.

The features used by these two models include all the previously described patient features in the GHRI dataset: age, gender, baseline PHQ-9 score, and CUIs from clinical text and coded data. We train each model on a randomly selected 80% of the GHRI patients satisfying the criteria for that model and test the model on the remaining 20%.

For both the training and test data, we exclude EHR data within 10 days prior to the start of the first treatment, to avoid including the period between diagnosis of depression and start of treatment. This is because our goal is to assess severity of depression from the patient's medical history alone, without incorporating the physician's initial evaluation at diagnosis.

In addition to predicting response to a particular category of treatment, we are also interested in identifying moderators of depression treatment—those patient characteristics that predict which treatment will perform better for a given patient. We use information gain to choose the 100 top predictors of a change of at least ±5 points in the PHQ-9 score after medication and psychotherapy. Then we perform a standard moderator variable analysis: we run logistic regression on these predictors, their corresponding interaction terms with the treatment variable, and a boolean treatment vector (1 for treatment with medication and 0 for treatment with psychotherapy). For example, abortion is one of these top 100 predictors, so we include the *abortion* variable and *treatment* variable, as well as *abortion* × *treatment*, which is 1 if the corresponding patient was both treated with medication and had an abortion mentioned in her clinical text, and 0 otherwise.

### Assessing severity

We also train and test a LASSO model to predict the baseline depression severity of GHRI patients, quantified by the PHQ-9 score at the start of the first treatment. Of the 5651 GHRI patients, 1014 have minimal or mild depression and 1301 have severe depression. Our model attempts to detect severe depression in patients (PHQ-9 score >19) (table 1). The model uses the same features as in predicting response to treatment. However, we exclude baseline PHQ-9 score because it is a direct measure of severity, which we are attempting to predict. The model is trained on a randomly selected 80% of the patients and tested on the remaining 20%. Similar to before, we exclude data within 10 days prior to initiation of treatment.

## Results

### Predicting diagnosis

**Figure 2:**
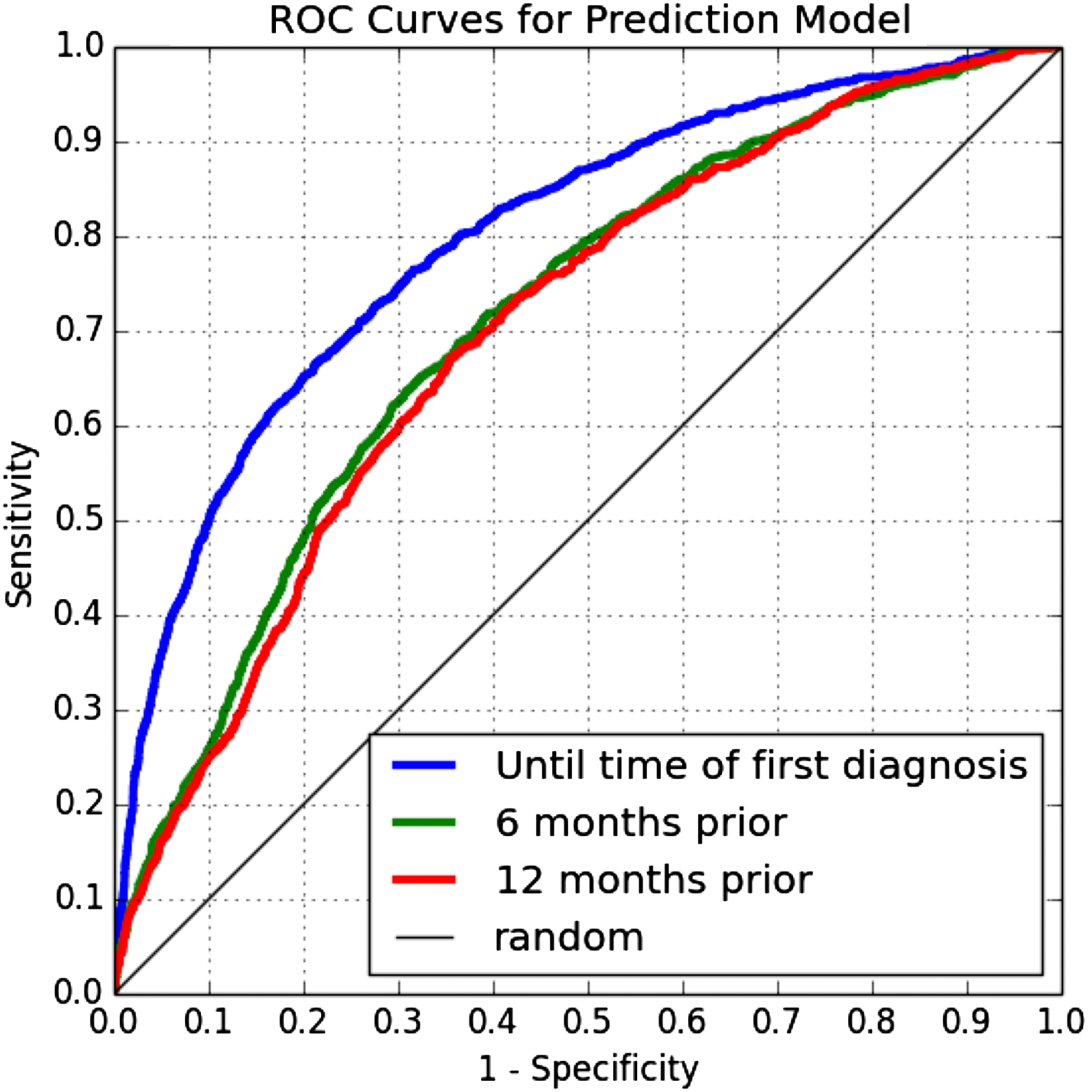
Receiver operating characteristic (ROC) curves for the model's performance on test data restricted to three cutoff points.

Using data for depressed patients up until the time of first diagnosis, the model for predicting diagnosis achieved an area under the receiver operating characteristic (AUC) curve of 0.800 (95% CI 0.784 to 0.815). This cutoff corresponds with a timely prediction. The AUC is 0.712 (95% CI 0.695 to 0.729) for the 6-month cutoff and 0.701 (95% CI 0.684 to 0.718) for the 1-year cutoff. In total, 553 out of the 10 600 features were used in this model. Figure 2 shows the receiver operating characteristic (ROC) curves for these three cutoffs, which display the full range of sensitivity and specificity values achieved by the model. For example, the ROC curves show that at 90% specificity (ie, 10% false positive rate), the model is able to predict a diagnosis of depression at approximately 50% sensitivity at the time of diagnosis and at 25% sensitivity 12 months prior to the time of diagnosis.

### Predicting response to treatment

As mentioned, the GHRI dataset contains 1576 patients whose depression severity reduced after they were treated with medication and 896 patients whose depression severity was unchanged or increased. Our model for predicting improvement after treatment with medication achieved an AUC of 0.661 (95% CI 0.607 to 0.715), using 133 features on average. Similarly, the dataset had 886 patients whose depression severity reduced after treatment with psychotherapy and 1515 patients whose severity remained unchanged or increased. Our model achieved an AUC of 0.749 (95% CI 0.706 to 0.792) for predicting improvement for these patients, using 193 features on average.

In both cases the most predictive feature was the baseline PHQ-9 score, and higher values corresponded with a worse outcome. We were unable to identify statistically significant moderators for differential response to medication versus psychotherapy.

### Assessing severity

Our model achieved an AUC of 0.718 (95% CI 0.671 to 0.764) for differentiating between baseline PHQ-9 scores of minimal/mild and severe, using 184 features on average.

## Discussion

Our results suggest the use of EHR data can improve the timely diagnosis of depression, which is associated with better prognoses when combined with prompt initiation of treatment.^4^ ^5^ Ideally, we are searching not only for models that can diagnose depression early to improve prognosis, but also for moderators that predict outcomes and enable personalized treatment. The latter requires significant work.

Diagnosing depression in primary care is challenging: Mitchell *et al*^1^ found primary care physicians have a sensitivity of 50% and a specificity of 81% in diagnosing depression. In comparison, at a specificity of 80% our model has a sensitivity of above 65% for predicting diagnosis at the original time of diagnosis, and nearly 50% for predicting it 6 months prior to the time of diagnosis. However, these diagnoses likely contain misdiagnoses (as Mitchell *et al* observed in other datasets), and thus do not correspond exactly to the patient having depression. If our model were trained on a gold standard with verified diagnoses, it would likely perform similarly, if not better, at predicting depression.

Our models for predicting improvement after treatment with medication and psychotherapy both detected baseline PHQ-9 score as the most predictive feature: a higher baseline PHQ-9 score, or more severe depression, was associated with a poorer outcome. This agrees with earlier studies associating severe depression with poorer treatment outcomes in general across different treatments^7^ ^13^ and argues for routine use of the PHQ-9 as an assessment instrument prior to initiating therapy.

To develop models that uncover moderator effects for personalizing treatment, and given the number of possible characteristics to consider (eg, comorbidities and prescriptions), much larger datasets are required. We may have been unable to identify statistically significant moderators because such moderators exist in non-EHR data (eg, genomics, environmental and lifestyle factors).

In learning models for moderator effects of treatments for depression, three things are clear: we need to accurately identify patients with (and without) depression, we need to consider the severity of the depression,^7^ ^13^ and we need large enough examples to guarantee the statistical power necessary when examining many possible moderators. Our work describes a method that reduces reliance on specific rating scales and thus could allow us to construct such retrospective cohorts across EHR systems more portably. Our model for differentiating patients with severe depression from those with minimal or mild depression had an AUC of 0.718. Thus, such a model for diagnosing and assessing severity of depression can help in building and assembling large EHR datasets from multiple sites for future analysis.

### Limitations

One limitation of our work is the accuracy of our annotation workflow in extracting disease and drug ingredient terms from clinical notes. Although prior validation efforts have indicated high accuracy, those estimates may change with a different target condition.^17^ Our work is also limited by the quality of our gold standard: that is, the PAMF depression and control cohorts. Although manually validated via random sampling, the labels (depressed vs non-depressed) we assign to patients may be incorrect, thus introducing inaccuracies and potential bias in both training and testing our model. For instance, certain patients in the control cohort may have depression but are never diagnosed with it, or some patients in the depression cohort may be misdiagnosed with depression. This may be due not only to the difficulty of diagnosing depression, but also deliberate misdiagnosis in the former case.^1^ ^30^ In addition, by limiting our depression cohort of PAMF patients to those who have taken antidepressants, we are including neither depressed patients who were treated solely with psychotherapy, nor those who chose to not undergo treatment. As a result, our depression cohort is potentially biased towards more severely depressed patients, since they required antidepressants as treatment.^14^ ^15^

To determine the sensitivity of our depression cohort construction process, we applied the selection criteria to 42 randomly selected patients from PAMF whose EHR progress notes were manually reviewed by experts to decide whether they had depression or not. We classify patients as depressed if they have a depression-related ICD-9 code in their medical history, as well as a depression term and an anti-depressive drug ingredient term in their clinical text. Based on these criteria, we achieved a precision of 100% and a sensitivity of 48% on classifying these 42 patients, on par with the results of other methods using structured data to identify depression.^31^

The time of first diagnosis calculated for the depressed patients is an approximation to the actual date at which the patient was first diagnosed with depression. The PAMF dataset we used also excludes notes from mental health professionals, due to privacy regulations. Inclusion of these notes may have improved the accuracy of our model for patients with these notes in their medical record. In addition, we acknowledge that predicting diagnosis of depression is not synonymous with predicting true onset of depression in patients.

Finally, our model for predicting diagnosis of depression can be converted to an electronic phenotyping model for identifying patients with depression. Having a regression model to identify patients for inclusion in studies may work better than ad hoc algorithms for electronic phenotyping and has broad applications, eg, for phenome-wide association studies.^32^ ^33^ In contrast to the existing model, a phenotyping model would not restrict depression patients’ EHR data and would also include depression-related ICD-9 features, disease terms, and drug terms. (These depression-related features were excluded from the prediction model because they were already used to define the depression and control cohorts.) With these highly relevant additional features, our model's performance should be on par with that of existing electronic phenotyping efforts for other disorders.^11^ ^34–36^

## Conclusion

We developed and assessed models that use EHRs for predicting the diagnosis and assessing the severity of depression. The model for predicting diagnosis uses ICD-9 codes, disease and drug ingredient terms extracted from clinical notes, and patient demographics as features to achieve an AUC of 0.70–0.80 for predicting a diagnosis of depression in patients, up to 12 months before the first diagnosis of depression. Even up to a year before their diagnosis of depression, patients show patterns in their medical history that our model can detect. These results suggest the use of EHR data can improve the timely diagnosis of depression, a disorder that primary care physicians often miss. In addition, our model for identifying patients with severe baseline depression achieved an AUC of 0.718 when compared against patients with minimal and mild depression. Use of such models may enable the merging of disparate EHR datasets to assemble datasets large enough to uncover moderator effects.

## Supplementary Material

Web supplement

Web supplement

Web supplement
