# Supplementary material for: Toward personalizing treatment for depression: predicting diagnosis and severity
Source: J Am Med Inform Assoc. 2014 Jul 2;21(6):1069–75. doi: 10.1136/amiajnl-2014-002733 (PMC4215055; doi:10.1136/amiajnl-2014-002733)
Supplement: Web supplement [file amiajnl-2014-002733-s1.pdf]

List 1: The 22 clinically relevant ontologies used in our optimized version of the NCBO Annotator.

Ontology Name	Source	Abbreviation
Anatomical Therapeutic Chemical Classification System	WHOCC	ATC
Current Procedural Terminology	UMLS	CPT
Coding Symbols for Thesaurus of Adverse Reaction Terms	UMLS	COSTART
US Food and Drug Administration Medical Devices	-	-
Healthcare Common Procedure Coding System	UMLS	HCPCS
Human Disease Ontology	OBO	DO
International Classification of Disease (ICD-10)	UMLS	ICD10
International Classification of Disease (ICD-9)	UMLS	ICD9
Logical Observation Identifier Names and Codes	UMLS	LNC
Medical Dictionary for Regulatory Activities	UMLS	MDR
MedlinePlus Health Topics<br>MEDLINEPLUS	UMLS	
Medical Subject Headings	UMLS	MSH
National Cancer Institute Thesaurus	UMLS	NCI
National Drug File	UMLS	NDFRT
Online Mendelian Inheritance in Man	UMLS	OMIM
Ontology of Adverse Events	OBO	OAE
RadLex	NCI	RADLEX
RxNORM	UMLS	RXNORM
Syndromic Surveillance Ontology	-	SSO
Systematized Nomenclature of Medicine-Clinical Terms	UMLS	SNOMEDCT
Universal Medical Device Nomenclature System	UMLS	UMD
World Health Organization Adverse Reaction Terminology	UMLS	WHO-ART
