# Supplementary material for: Toward personalizing treatment for depression: predicting diagnosis and severity
Source: J Am Med Inform Assoc. 2014 Jul 2;21(6):1069–75. doi: 10.1136/amiajnl-2014-002733 (PMC4215055; doi:10.1136/amiajnl-2014-002733)
Supplement: Web supplement [file amiajnl-2014-002733-s2.pdf]

List 2: Depression disorder terms. Patients in the depression cohort must contain at least one of these terms in their clinical text.

adjustment reaction with brief depressive reaction
adjustment reaction with prolonged depressive reaction
agitated depression
atypical depression
atypical depressive dis
brief depressive adjustment reaction
childhood depression
chronic depression
chronic depressive personality disorder
chronic recurrent major depressive disorder
depress psychosis-mild
depress psychosis-severe
depression
depression aggravated
depression agitated
depression mental
depression neurotic
depression nos
depression postmenopausal
depression reactive
depression suicidal
depression, endogenous
depression, neurotic
depression, post-partum
depression, postpartum
depression, reactive
depression, refractory
depression, unipolar
depressive dis
depressive disorder
depressive disorder nec
depressive disorder nos
depressive disorder, major
depressive disorder, nos
depressive disorder, not elsewhere classified
depressive disorders
depressive episode
depressive illness
depressive psychosis-mod
depressive reaction
depressive type psychosis
disorder, depressive
disorder, dysthymic

disorder, major depressive
disorder, seasonal affective
disorders, major depressive
dysthymia
dysthymic dis
dysthymic disorder
dysthymic disorders
endogenous depression
grade 1 depression
involutional depression
major depression
major depression in complete remission
major depression in partial remission
major depression in remission
major depression, single episode
major depressive disorder
major depressive disorder, nos
major depressive disorder, recurrent
major depressive disorder, recurrent episode
major depressive disorder, recurrent episode, in full remission
major depressive disorder, recurrent episode, in partial or unspecified remission
major depressive disorder, recurrent severe without psychotic features
major depressive disorder, recurrent, mild
major depressive disorder, recurrent, moderate
major depressive disorder, single episode
major depressive disorder, single episode in full remission
major depressive disorder, single episode, in partial or unspecified remission
major depressive disorder, single episode, in partial remission
major depressive disorder, single episode, mild
major depressive disorder, single episode, moderate
major depressive disorder, single episode, unspecified
major depressive disorders
major depressive illness
masked depression
melancholic depression
menopausal depression
mild depression
mild major depression
mild postnatal depression
mild recurrent major depression
minor depressive disorder
moderate depression
moderate major depression
moderate major depression, single episode
moderate recurrent major depression
mood disorder, seasonal

mood disorders, seasonal
neurotic depression
post natal depression
post partum depression
post stroke depression
post-natal depression
post-partum depression
postmenopausal depression
postnatal depression
postoperative depression
postpartum depression
prolong depressive react
prolonged depressive adjustment reaction
psychotic depression
reactive depression
reactive depression (situational)
reactive depressions
recurr depr psychos-mild
recurr depr psychos-mod
recurrent depression
recurrent major depression
recurrent major depression in remission
recurrent major depressive episode
recurrent major depressive episodes
refractory depression
seasonal affective disorder
seasonal affective disorders
seasonal depression
seasonal mood disorder
secondary dysthymia
severe depression
severe major depression with psychotic features
severe postnatal depression
severe recurrent major depression with psychotic features
single episode of major depression
single episode of major depressive disorder
single major depressive episode
single major depressive episode, moderate
suicidal depression
treatment resistant depression
unipolar depression
unipolar depressions
