# Supplementary material for: Toward personalizing treatment for depression: predicting diagnosis and severity
Source: J Am Med Inform Assoc. 2014 Jul 2;21(6):1069–75. doi: 10.1136/amiajnl-2014-002733 (PMC4215055; doi:10.1136/amiajnl-2014-002733)
Supplement: Web supplement [file amiajnl-2014-002733-s3.pdf]

List 3: Anti-depressive drug ingredients. Patients in the depression cohort must contain at least one of these terms in their clinical text.

alprazolam
amitriptyline
amoxapine
aripiprazole
buspirone
citalopram
desipramine
desvenlafaxine
duloxetine
escitalopram
fluoxetine
imipramine
isocarboxazid
lorazepam
maprotiline
mirtazapine
nefazodone
nortriptyline
olanzapine
paroxetine
phenelzine
probenecid
protriptyline
quetiapine
sertraline
tranylcypromine
trazodone
trimipramine
venlafaxine
